# Scoping review of the morphology and anthropometry of Tessier craniofacial clefts numbers 3 and 4

**DOI:** 10.1186/s13643-019-0951-6

**Published:** 2019-02-04

**Authors:** Abiola Omodan, Pamela Pillay, Lelika Lazarus, Anil Madaree, Kapil Satyapal

**Affiliations:** 1 Department of Clinical Anatomy, School of Laboratory Medicine and Medical Sciences, University of KwaZulu-Natal, Durban, South Africa; 2 Department of Plastic and Reconstructive Surgery, University of KwaZulu-Natal, Durban, South Africa

**Keywords:** Craniofacial clefts, Tessier number 3, Tessier number 4, Morphology, Anthropometry

## Abstract

**Background:**

In 2016, WHO reported a death rate of 303,000 newborns before 4 weeks of age due to congenital anomalies. Those that survive congenital anomalies may have long-term disabilities which may have significant impacts on the individual, their families, the healthcare system, and societies. Tessier craniofacial clefts numbers 3 and 4 are congenital anomalies that result in a partial or total defect of craniofacial tissues thereby seriously influencing the patient’s appearance and impair normal functioning. Therefore, understanding these defects is paramount to relieving the burden caused by this disability. The objective of this review was to examine the literature on the understanding of the knowledge of morphology and anthropometry of Tessier craniofacial clefts numbers 3 and 4 so that areas yet to be fully understood by research can be mapped out for future research.

**Methods and analysis:**

A scoping review for literature on patients who have Tessier craniofacial clefts numbers 3 and 4 was conducted. Relevant studies from 1976 to the present were identified. The following databases were searched for peer-reviewed literature viz., PubMed, MEDLINE, EBSCOhost, Google Scholar, and the Cochrane library. The study selection was guided by the eligibility criteria. A data table was designed to extract information from the literature. The result of this study was reported using the Preferred Reporting Items for Systematic reviews and Meta-analyses (PRISMA). The quality of the included studies was assessed using the Mixed Method Appraisal Tool (MMAT).

**Result:**

Thirty-three studies met the inclusion criteria. The majority of the studies included were conducted in middle-income countries (54.5%) and some in high-income countries (45.5%); none was recorded from low-income countries. The total available sample size from the studies was 120 with a dominant male population of 67 (55.8%) and female 53 (44.2%). The majority (97%) of the studies reported on the knowledge of morphology while 12.1% of the included studies reported on anthropometry. Of the 33 included studies, 32 scored the highest quality (76–100%) from the quality assessment.

**Discussion:**

The findings from this review show evidence of the knowledge of morphology and the knowledge of anthropometry of Tessier craniofacial clefts numbers 3 and 4. However, these knowledges have not translated to universally recognized ways of repairing and documenting these clefts due to the sparse amount of studies on Tessier craniofacial clefts numbers 3 and 4.

**Electronic supplementary material:**

The online version of this article (10.1186/s13643-019-0951-6) contains supplementary material, which is available to authorized users.

## Background

Congenital craniofacial cleft deformity is a partial or total defect of the soft tissue, bone, or a combination of these tissues in the craniofacial region. The severity of this deformity can range from relatively minor skin and soft tissue deformities to major bony abnormalities or defects of the cranial, orbital, or facial skeleton [1]. Craniofacial clefts can occur anywhere in the craniofacial area and may seriously influence the patient’s appearance and impair normal functioning [1]. The incidence of the rare facial clefts is between 1.43 and 4.85 per 100,000 births [2]. Craniofacial anomalies affect a significant proportion of society with the ratio varying considerably by geographical area and ethnic grouping [3]. The costs of these anomalies in terms of morbidity, healthcare, emotional disturbance, and social and workplace exclusion are considerable for affected individuals, their families, and society [3].

According to the World Health Organization (WHO), 303,000 newborns die within the first 4 weeks per year worldwide from congenital anomalies, of which Tessier craniofacial clefts numbers 3 and 4 are included [4]. Since one of the Sustainable Development Goals (SDG) of the World Health Organization (WHO), as well as South Africa, is to ensure healthy lives and promotion of well-being at all ages, knowledge and understanding how to solve this problem are critical for these anomalies [5].

Rare craniofacial clefts pose the most significant reconstructive challenge to the craniofacial surgeon today because of their variability and complexity [6]. Facial cleft surgery publications are sparse due to the rarity of the disorders, and consensus has yet to develop on standardized landmarks, reference measurements, and principles of repair [7]. Understanding the soft tissue and skeletal deformity is basic to any reconstructive surgical procedure of the face [2]. There is little evidence in the literature to suggest that much is being done to address this issue.

Therefore, the objective of this review is to map out evidence of the knowledge of morphology (particular forms in which these clefts present) and anthropometry (description of these clefts using measurements or the use of some form of measurements in their repair) of Tessier craniofacial clefts numbers 3 and 4. Researchers in the field of craniofacial anomalies may benefit from the findings of this review as it highlights areas still undiscovered in the pursuit of understanding the variant anatomy of Tessier craniofacial clefts numbers 3 and 4.

## Methods

This study on the knowledge of morphology and the knowledge of anthropometry of Tessier craniofacial clefts numbers 3 and 4 is a part of a larger study looking at “Understanding Tessier craniofacial clefts numbers 3 and 4: A scoping review,” which is a part of a study looking at the “Anatomical classification of Tessier craniofacial clefts numbers 3 and 4 in a select South African population.”

A scoping review research method is defined as a “type of research synthesis that aims to map the literature on a particular topic and provide opportunity to identify gaps to guide future studies.” [8].

This scoping review began with the establishment of a research team consisting of individuals with expertise in epidemiology and research synthesis [9]. The team advised on the broad research question to be addressed and the overall study protocol, including identification of search terms and selection of databases to search. The methodology for this scoping review was based on the framework outlined by Arksey and O’Malley (2005) and ensuing recommendations made by Levac and colleagues (2010) [9, 10]. The review included the following five key phases: (1) identifying the research question; (2) identifying relevant studies; (3) study selection; (4) charting the data; and (5) collating, summarizing, and reporting the results. A detailed review protocol can be obtained from the primary author upon request.

### Research question

This review was guided by the main question “What types of research on Tessier craniofacial clefts number 3 and 4 have been reported?” and a sub question “In which countries (High, Middle OR low income) are research on Tessier craniofacial clefts number 3 and 4 being reported?”. We applied the PCC (Population, Concept and Context) framework to determine the eligibility and appropriateness of the primary research question. The results of the scoping review were reported using the PRISMA (Preferred Reporting Items for Systematic reviews and Meta-analysis) guidelines. See Additional file 1.

### Data sources and search strategies

The initial search was implemented on 29 November 2017, in four electronic databases: viz., Google Scholar, EBSCOhost (Academic search complete, Educational source, Health source, Nursing/Academic, Medline with full text, Medline), PubMed, and the Cochrane library. The databases were selected to be comprehensive. No limits were made on language; however, there was a date limit (1976 and above, which is the year that the current accepted classification by Tessier was published). The search query consisted of terms considered by the authors to describe the scoping review and its methodology: Tessier clefts, Tessier number 3, Tessier number 4, Tessier number 3 and number 4 morphology, Treatment, Treatment outcome, and anthropometry. The search query was tailored to the specific requirements of each database. The entire literature search strategy, reflecting dates, database, search terms, and the results were documented.

### Citation management

All citations were imported into EndNote X7, and duplicate citations were removed manually with further duplicates removed when found later in the process before they were subsequently used in title and abstract screening and data characterization of full articles.

### Eligibility criteria

A set of questions was used to assess the relevance of studies identified in the search. Studies were eligible for inclusion if they reported on Tessier craniofacial clefts number 3 or number 4, if they were done in 1976 and later, and if the studies had information on the morphology and anthropometry of Tessier craniofacial clefts numbers 3 or 4. Studies that were not primarily on Tessier craniofacial clefts number 3 or number 4 were excluded as well as studies done pre-1976.

### Title, abstract, and full-article relevance screening

For the first level of screening, one member of the team screened the titles from the databases and exported eligible articles to an Endnote library ready for abstract screening. For the second level, an abstract relevance screening form was developed by the authors and reviewed by the research team. Two members of the research team (AO and DK) independently screened the abstracts. Any disagreements at this level of the research necessitated both reviewers to meet for discussion until a common consensus was reached. All citations deemed relevant after title and abstract screening were procured for subsequent review of the full-text article.

For articles that could not be obtained through institutional holdings available to the authors, attempts were made to contact the source author or journal for assistance in procuring the article. The third level of screening involved creating a full-article screening form, and two members of the team (AO and TS) independently screen the full articles. Disagreements at this stage were however resolved by involving a third reviewer (TMT). The degree of agreement for the full-article screening was calculated with the overall kappa being 0.989, where a kappa of greater than 0.8 is considered to represent a high level of agreement [11] (see Additional file 1). Reviewers met throughout the screening process to resolve conflicts and discuss any uncertainties related to study selection [9].

### Quality assessment of individual studies

All the 33 included primary studies underwent methodological quality assessment using the Mixed Methods Appraisal Tool (MMAT)—version 2011 [12] (see Additional file 2). A form was designed by the authors, and it was then piloted by the research team before it was used on the articles. The studies were assessed in the following domains: the clarity of the research question, relevance of the sampling strategy to the research question, appropriateness of the measurements, and appropriate representation of the population under study. An overall quality percentage score for each of the included studies was calculated, and the scores were interpreted as low quality (≤ 50%), average quality (51–75%), and high quality (76–100%).

### Data characterization

A form was developed by the authors to confirm relevance and to extract study characteristics such as author and date, title, main objective, knowledge of morphology, knowledge of treatment, knowledge of treatment outcome, knowledge of clinical spectrum, knowledge of anthropometry, most significant outcome, study design, other significant findings, country of the study, and high- or middle/low-income country. Other information such as the percentage of male or female and age range of the population was extracted. The research team reviewed this form and slight modifications were made before use.

### Data summary and analysis

The data were compiled in a single spreadsheet and imported into Microsoft Excel 2010. Content analysis of each emerging theme was done. The emerging themes identified for this paper included the knowledge of anthropometry and the knowledge of morphology. These themes were extracted from all studies that were included.

### Patient and public involvement

Patients were not involved in this study.

## Results

**Fig. 1:**
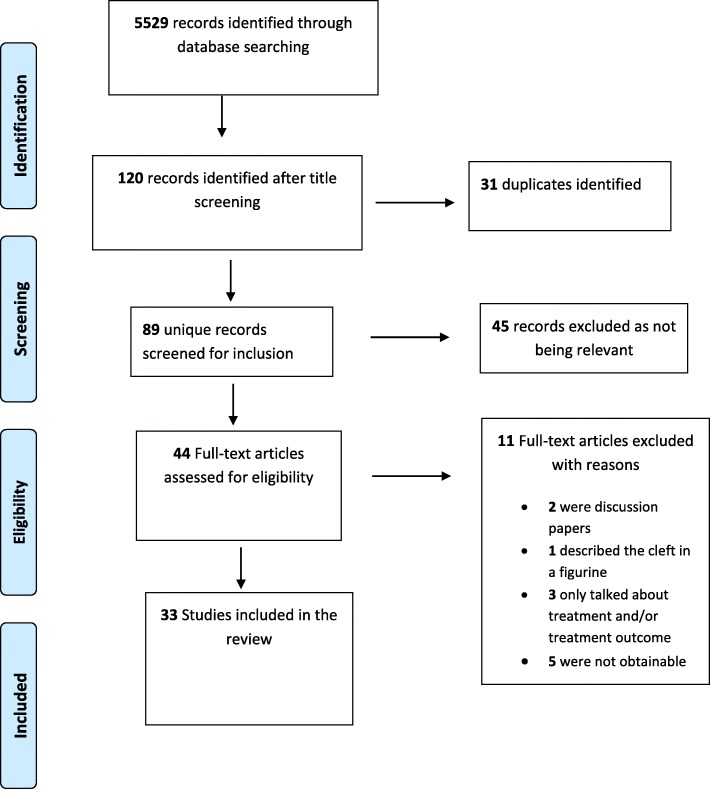
PRISMA flowchart of study selection process

The original search conducted in November 2017 yielded 5529 potentially relevant citations. After deduplication and relevance screening, 44 citations met the eligibility criteria based on the title and abstract and the corresponding full-text articles were procured for review. After data characterization of the full articles, 33 studies remained and they were included in the analysis (see Additional file 2). The flow of articles through identification to final inclusion is represented in Fig. 1.

Of the 11 articles excluded at full-article screening, five articles could not be procured and thus were not included in the review [13–17]. Two articles were discussion papers [18, 19]. Bartlett discussed the study of [6] which is one of the included studies, while Resnick and Kawamoto discussed the morphology and treatment. Three articles were not relevant as they did not illustrate evidence of the knowledge of anthropometry nor the knowledge of morphology which are the themes for this paper [20–22]. Aleman and Martinez described the cleft in an ancient figurine [23].

### Characteristics of included studies

**Fig. 2:**
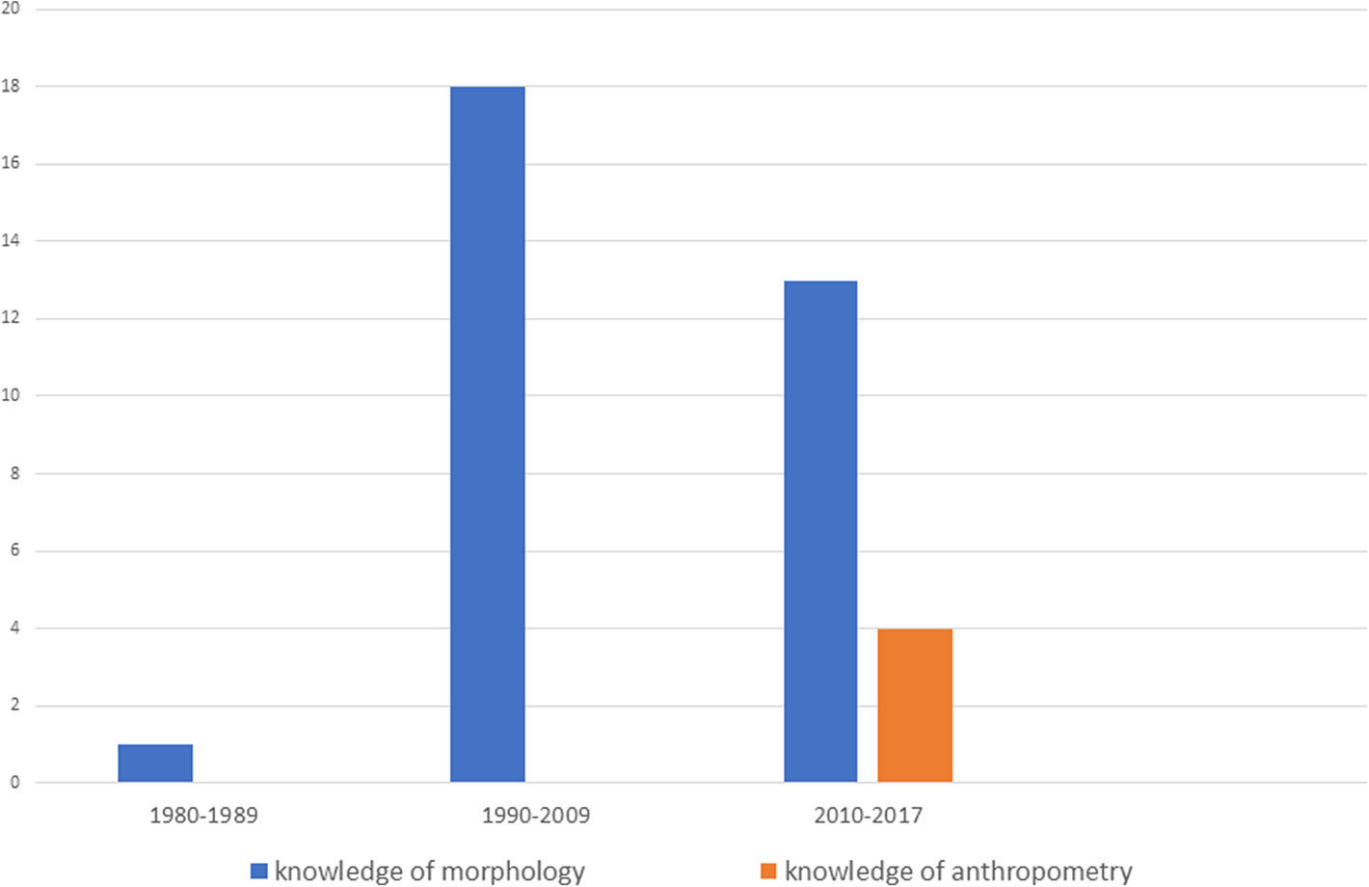
Graph showing timeline of advent of knowledge of morphometry and anthropometry in the literature in the past 3 decades. *X*-axis displays the year and *y*-axis illustrates number of articles

Table 1 shows the characteristics of the included studies while Fig. 2 shows a graphical representation of the advent of the knowledge of morphology and anthropometry in literature pertaining to Tessier craniofacial clefts number 3 and number 4. The majority (54.5%) of the included studies were done in middle-income countries [24–41], 45.5% in high-income countries [6, 7, 42–54], and none was carried out in low-income countries. All included studies were published between 1980 and 2017. The total available sample size from the studies was 120 with a dominant male population of 67 (55.8%) and female 53 (44.2%). All the included studies were either case reports or case series. The majority (97%) of the studies reported on the knowledge of morphology [7, 24–54] while 12.1% of the included studies reported on anthropometry [6, 7, 40, 45].

**Table 1:** Characteristics of articles included

Author/date	Percentage (male)	Percentage (female)	Age of population	Type of cleft	Aim of study	Main outcome
Ahmad Muhsin Mohammad Nor, 2016	100%		1 day old	FC 4	The aim of this presentation is to report the multidisciplinary sequence of procedures to manage Tessier number 4 facial cleft	A multi-disciplinary approach in managing such patient is paramount due to the complexity. Besides the obvious issues, the psychosocial aspect of this matter must also be looked into
Shahin Abdollahi Fakhim et al., 2012		100%	6 months old	FC 4 and 5	Was to present a patient with bilateral numbers 4 and 5 Tessier cleft lip with unilateral complete cleft palate and surgical approach on her	We recommended early repair using autogenous tissues and as minimal disposal of the healthy tissues as possible
Akoz et al., 1995	100%		4 months old	FC 4	To report a case of Tessier no.4 cleft and their surgical approach to repairing it	Using a technique of surgery that preserved maximal amount of soft tissue with conjunctival and lower lid reconstruction utilizing a flap from the medial cleft ridge
Allam et al., 2014	70%	30%	6 weeks old to 20 years old	FC 3	Considering the rarity of the Tessier number 3 cleft, the objective was to review one of the largest series in the literature describing a single surgeon’s experience in treating this complex facial cleft	As these clefts can be variable in presentation, each treatment approach must be individualized to the patient and their needs
Nivaldo Alonso et al., 2008	66.7%	33.3%	1 day old to 25 years old	FC 4	The present article aims to describe different clinical features evidenced in 21 cases of this malformation, discussing a 20-year experience with and evolution of its surgical treatment	According to our reconstructive experience, the great majority of Tessier no. 4 facial clefts may be appropriately treated using local flaps. Classic techniques are extremely useful and can offer good functional and esthetic outcomes
S. M. Balaji, 2017	100%		18 months old	FC 4	This article presents a rare case of an 18-month-old baby with bilateral Tessier no. 4 clefts and its successful rehabilitation	Early repair using autogenous tissues and minimal discarding of healthy tissues as much as possible is recommended
F. Bodin et al., 2005		100%	4 months old	FC 3, 7, and 11	We report a case of right associated Tessier no. 3, 7, and 11 craniofacial clefts with cardiac malformation	The case we report is a unique association of severe hemifacial microsomia and complete oro-naso-ocular cleft. To our knowledge, this association has not been previously reported
Chen et al., 2011	N/A	N/A	N/A	FC 3 and 4	Considering the rarity of the Tessier number 3 cleft, the objective was to review one of the largest series in the literature describing a single surgeon’s experience in treating this complex facial cleft	The “midface rotation advancement” concept and technique give rise to esthetically favorable results both in primary and in secondary reconstructions. This technique avoided significant scarring with poor skin color matching and unnatural facial expressions associated with the interdigitating skin flap technique
Atilla Coruh et al., 2003	50%	50%	15 days to 7 years	FC 4	This article presents two cases of Tessier no. 4 clefts, one unilateral and the other bilateral, and discusses the problems encountered during their surgical and postoperative managements	If the soft tissue deficiency is severe, conventional techniques using flaps or Z-plasties, which are designed to replace the missing tissues, are far from being ideal. Mustarde cheek flaps for lower eyelid reconstruction may be an alternative for extensive facial clefts
Renato da Silva Freitas et al., 2009	43%	57%	1 day old to 30 years old	FC 3	The objective was to review the functional outcome and esthetic results of the different techniques applied for each case	We have treated 21 patients with Tessier number 3 cleft at 2 craniofacial centers. Eyelid, nose, and upper lip deformities should be treated in sequential stages, positioning the medial canthus, ala, and upper lip, using the contralateral side as the reference
Mohd Ashraf Darzi et al., 1993	66.7%	33.3%	3 months old to 3 years	FC 3, 4, 5, and 9	To adequately examine the occurrence of oblique clefts, the medical community must be aware of the problem and new cases should be presented. On the basis of clinical radiologic and surgical examinations, soft tissue and skeletal disruptions of three patients with the most rare craniofacial clefts (Tessier 3, 4, 5, and 9) are presented	Kawamoto reported Tessier number 5 cleft to be the least frequently observed oblique facial cleft. Our case three is the third bilateral and overall, the ninth case of Tessier number 5 clefts reported in world literature
E. Gawrych et al., 2010		100%	2 weeks old	FC 3	This report presents a patient with a right-sided oblique cleft extending through the upper lip, the alar groove, and the lower palpebra accompanied by a left-sided complete cleft lip and palate. Hypertylorism and bilateral microphthalmia as well as flexion wrist contractures were also present	The findings of this report demonstrate the wide variability in the pattern of presentation of oblique facial clefts caused by aberrant tissue bands
Alcir Giglio et al., 2008	25%	75%	5 months to 8 years	FC 3 and 4	Considering the rarity of the Tessier number 3 cleft, the objective was to review one of the largest series in the literature describing a single surgeon’s experience in treating this complex facial cleft	The rotation and advancement flap of the cheek is a safe technique that may present satisfactory results in the treatment of rare craniofacial nos. 3 and 4 clefts
Ugur Horoz et al., 2016	50%	50%	1 to 12 years old	FC 4	The present study presents a new lip-rescue flap technique as an alternative approach for reconstructing Tessier no. 4 facial clefts	We applied our lip-rescue flap surgically on 4 patients with Tessier no. 4 facial clefts and found that the design rendered adequate tissue support and provided acceptable functional and esthetic results. Because it achieves more tissue support, we recommend using this lip-rescue flap as a reconstruction method in appropriate patients of Tessier no. 4 facial clefts
Boris Laure et al., 2009	100%		26 years old	FC 4	We report a case of a complete bilateral Tessier number 4 cleft and our approach to surgical correction. We analyze the patient’s treatment plan over a 26-year follow-up period	These rare facial clefts should be treated with the same surgical management principles as the more common lip and palate clefts
Longaker et al., 1996	33.3%	66.7%	2 to 5 months	FC 4 and 1/13, 2/12, 3/11	We present two cases of Tessier no.4 clefts and one case of a multiple clefted (Tessier nos. 1/13, 2/12, 3/11) child with the typical contracted oculo-alar and oculo-oral distances. Reconstruction with a superiorly based nasolabial flap transposed 90 degrees under the eye was performed in all three as a primary procedure	The preceding reconstruction approach provided early protection of the eye, better position of the medial canthus, reconstitution of the bony orbit, and immediate improvement in facial appearance
Madaree et al., 1992	100%		6 weeks old	FC 3	A method of correction of an incomplete no. 3 facial cleft in an infant is presented. It is compared with previously described repairs, and its advantages are outlined	We feel that our inferiorly based transposed paranasal flap is a preferable method of filling the defect above the released alar rim
Maeda et al., 2014		100%	1 day old	FC 3 and 4	Here we present the first case of a girl born with a combined anomaly of Tessier clefts 3 and 4 with severe bilateral cleft lip, a displaced premaxilla, and three-dimensional underdevelopment of the hard and soft tissues of the maxilla and zygoma	We report an extremely rare case of a combined anomaly of Tessier clefts 3 and 4, which is, to our knowledge, the first case described in the English literature
Mishima et al., 1996	66.7%	33.3%	N/A	FC 3 and 4	This paper describes three cases of oblique facial cleft, one of which was obviously accompanied by an amnion rupture sequence. Of the other two cases, one was not affected by an amnion rupture sequence, while the other may have been	The cause can be adjudged to embryological development. Among our cases, case 3 displayed conditions typical of the amnion rupture sequence, and an amniotic band attached to an encephalocele was also detected
Mishira et al., 2009	28.6%	71.4%	1.5 to 21 years	FC 3 and 4	To overcome this problem and provide a ground rule for surgical management of such cases, we propose an easier format with a “split approach” of the affected areas	Also, surgeons are often faced with complexities like the ideal age for surgical intervention and methods to ensure minimal scars in these cases. In this article, we have tried to address these issues and have attempted to provide guidelines to manage such cases effectively on the basis of our experience of seven cases of Tessier cleft types 3 and 4 in their unilateral and bilateral forms
Morgan et al., 2016	100%		N/A	FC 3	The authors describe a method of correcting incomplete unilateral Tessier 3 cleft based on the principles of anthropometric techniques, based on identifiable landmarks and anthropometric measurements that are compared with contralateral unaffected anatomy or population means and tracked over time to assess impact on growth	We present a patient with a good long-term postoperative result based on anthropometric methods to reconstruction. We feel this initial technique along with documentation of subsequent procedures can help provide a more reproducible form of reconstruction of the soft tissues in this rare patient population
Porttier-Marriet et al., 2008		100%	6 months old	FC 4	We describe the case of a 9-month-old girl with a complete bilateral facial cleft. On the right cornea protruded a hard lesion, a corneal staphyloma	We describe the case of a 9-month-old girl with a complete bilateral facial cleft. On the right cornea protruded a hard lesion, a corneal staphyloma. We described the 3 primary surgical steps used to restore the possibility of satisfactory feeding, to promote language acquisition, and to protect vision in the non-affected eye
Reddy et al., 2014	66.7%	33.3%	2 to 11 years old	FC 2 and 3	We present two surgical options to repair such facial clefts.	We have been able to demonstrate that nasal dorsum rotation flaps were a viable option for treating the nasal defects of Tessier no. 2 facial clefts. Similarly, FENTF were a viable option to treat the nasal defects of Tessier no. 3 facial clefts.
A. Rintala et al., 1980	36.4%	63.6%	1 day old to 58 years old	FC 3, 4, 5, 7, 8, and 9	Explore cases of oblique facial clefts in the center	All patients represent different types of clefts, and in most cases, they are associated either with other facial defects or with defects of other developmental fields. There is a slight over representation of females (7:4)
Sari et al., 2003	100%		8 months old	FC 4	A patient with a Tessier number 4 cleft is presented, whose bony defect was obliterated with autogenous iliac bone graft chips and soft tissue reconstruction was performed with multiple Z-plasty flaps	Postoperative clinical and radiological results demonstrate fine healing and good cosmesis. Although controversy still exists about the treatment of facial clefts with early bone grafts, advantages of performing both bony and soft tissue reconstructions in a single session make this treatment a good alternative with satisfactory clinical and radiological results
Sessena et al., 2011		100%	1 day old	FC 3	The authors present a “step-by-step” solution of the malformation pointing out the limitations of the surgical procedures they used and the goals they wanted to obtain	The authors report an extremely rare case of a Tessier 3 cleft associated with bilateral anophthalmia, which is, as far as they know, the first one described in the English literature
Spolyar et al., 2015	100%		3 to 7 months	FC 3 and 4	Authors propose pre-surgical orthopedic correction for naso-oro-ocular clefts and a novel surgical option for Tessier no. 3 cleft	Presurgical assistance facilitates comprehensive repair of the severe facial clefts, even with single-stage primary defect repair during infancy. Lengthening of the ala base-canthal distance is a key achievement, and it can be addressed by performing a frontonasal flap extended with a myocutaneous upper lid flap
Tokioka et al., 2005	50%	50%	1 day old	FC 4	In this report, two cases with Tessier no. 4 cleft, which were treated with the cheek advancement flap technique, are presented	Our results indicated that by using the cheek advancement flap technique, the soft tissue deficiency of the lower eyelid was not satisfactorily reconstructed. It is suggested that any single flap is not enough for the eyelid reconstruction in such a wide cleft as in our cases. Correction with the other local flaps will be planned in the near future. Tissue expansion or free tissue transfer are good alternatives for soft tissue reconstruction
Uemura et al., 2004	100%		6 years old	FC 3	A composite Z-plasty to treat recurrence of cicatricial ectropion of the lower eyelids in Tessier 3 cleft is described	Composite Z-plasty is a convenient surgical method suitable for scar contracture of tissues with free margins, such as the eyelid, nostril rim, and auricular helix, from which support tissue and covering skin tissue must be harvested. Composite Z-plasty should be considered in treatment planning for ectropion
Wenbin et al., 2006	100%		1 day old to 2 years old	FC 3	Tessier 3 cleft with clinical anophthalmia is one of the rarest craniofacial clefts, and hence little has been published about its management and treatment. This article presents two cases of Tessier 3 cleft with clinical anophthalmia	
Wu et al., 2012		100%	20 months old	FC 3	A Uighur girl with severe bilateral Tessier 3 clefts and associated orofacial deformities is described here, and a novel protocol for clefts of this severity and rarity is presented. This study focuses particularly on describing the surgical procedures and techniques	The wide facial and palatal clefts were completely closured and the defective nasal ala and the dislocated medial canthi effectively reconstructed. The patient had an acceptable facial appearance with inconspicuous scars and natural facial expression. The outcomes of these operations were functionally and esthetically satisfactory
Xu et al., 2015	100%		1 day old to 6 months old	FC 3	In this paper, we report two extremely rare cases of simultaneous Tessier number 3 cleft, contralateral cleft lip, and signs of amniotic band syndrome	We report two extremely rare cases of Tessier number 3 cleft with contralateral cleft lip and signs of amniotic band syndrome. From these two cases, we may confirm that amniotic bands are the most probable cause of the Tessier number 3 cleft. Treatment of the Tessier number 3 cleft should be individually designed based on the severity of the deformities
Sigler et al., 2004	100%		6 months old	FC 2, 3, and 7	A unique case of a unilateral partial Tessier no. 7 cleft accompanied by nos. 2 and 3 clefts along with a single median lip pit is presented	After an extensive review of the literature, we found that unilateral transverse facial cleft along with unilateral CL/P and a median LP to our knowledge has never been describe

### Quality of evidence from included primary studies

All the 33 primary studies underwent methodological quality assessment using the Mixed Methods Appraisal Tool (MMAT)—version 2011 [12]. The studies were assessed based on all the categorized domains. Of the 33 included studies, 32 scored the highest (76–100%) [24–51, 53, 54]. The last one was not assessed further after scoring a “No” in the first question determining if there was a clear research question [52].

### Themes from included studies

#### Knowledge of anthropometry of Tessier craniofacial clefts number 3 and number 4

Four of the 33 (12.1%) included studies showed varying levels of the knowledge of anthropometry in discussing Tessier craniofacial clefts number 3 and number 4. Two of these studies showed the knowledge of anthropometry in the surgical reconstruction of the defects [6, 7] while the other two displayed the knowledge of anthropometry in defining the clefts and then reconstruction [40, 45].

- Knowledge of anthropometry in cleft definition and reconstruction

Two of the included studies showed evidence of the knowledge of anthropometry not only in the reconstruction of the defects but also initially in defining the clefts [40, 45]. One of the studies was done in a high-income country (USA) and the other in a middle-income country (China). In their study, Wu et al. aimed at describing a novel protocol for these clefts and attributed the satisfactory functional and esthetic outcomes to the novelty of the procedure [40]. These studies show a lack of literature on the emerging technique of anthropometry in the correction of defects of Tessier craniofacial clefts numbers 3 and 4.

(b) Knowledge of anthropometry in surgical reconstruction

Two studies showed the knowledge of anthropometry during surgical reconstruction of the defects in Tessier craniofacial clefts number 3 and number 4 [6, 7]. Both studies were carried out in high-income countries (Taiwan, USA). In a study by Chen and colleagues aimed at reviewing one of the largest series in the literature describing a single surgeon’s experience in treating this complex facial cleft, findings showed that previous treatment options paid little attention to the anatomical repair of the affected facial musculature which has led to sub-optimal results with conspicuous facial scars, poor color matching of the cheek and nasal flaps, and unnatural facial expression [6]. The study by Morgan et al. described a method of correcting incomplete unilateral Tessier 3 cleft based on the principles of anthropometric techniques showing that consensus has yet to be developed on standardized landmarks, reference measurements, and principles of repair due to sparse publications [7]. Evidence from these studies documented that knowledge of anthropometry is key to a satisfactory outcome in the surgical management of these clefts and there is a scarcity of publication on this knowledge, and this has made arriving at a consensus on standardized landmarks, reference measurements, and principle of repair difficult thus far.

#### Knowledge of morphology

Thirty-two (97%) of the included studies showed evidence of the knowledge of morphology of Tessier craniofacial clefts numbers 3 and 4 [7, 24–54]. Evidence shows that the knowledge of morphology was expressed by describing the clefts as complete, while five of the studies (15.6%) described a form of the cleft as incomplete [26, 27, 33, 34, 41].

- Knowledge of morphology of complete clefts

Thirty-one (96.9%) of the studies that showed the knowledge of morphology showed evidence of knowledge of a complete cleft [7, 24–32, 34–54]. Alonso et al., in their study which was on the different clinical features in 21 cases of number 4 cleft, described the complete cleft as consisting of a cleft lip, lateral to the Cupid’s bow, which crosses superiorly up to the lower eyelid, decreasing the oro-ocular distance [28]. While Allam et al., in a study reviewing one of the largest series of number 3 clefts in the literature, described the complete number 3 cleft as extending from the philtrum of the lip to the medial canthus of the eye with the foreshortening of this distance with affectation of the nasal ala [27]. Evidence from these studies show that there is a generally acceptable knowledge of the morphology of a complete cleft of Tessier craniofacial clefts numbers 3 and 4.

(b) Knowledge of morphology of incomplete clefts

Five (12.1%) of the 32 studies that reported on the evidence of the knowledge of morphology showed evidence of incomplete cleft [26, 27, 33, 34, 41]. In their studies, Giglio and colleagues who aimed at reviewing a large collection of the rare cleft number based on a single surgeon’s perspective and Madaree et al., whose aim was to describe a method of correction of an incomplete number 3 cleft while comparing with previously documented methods, described the incomplete cleft as sparing the lip [33, 34]. Evidence from these studies show that there is a paucity of literature on the morphology of the incomplete Tessier craniofacial clefts numbers 3 and 4.

## Discussion

This study sets out to map out evidence of the knowledge of morphology and the knowledge of anthropometry of Tessier craniofacial clefts numbers 3 and 4. A total of 33 unique articles were found that included the evidence of morphology and anthropometry of Tessier craniofacial clefts numbers 3 and 4. The result of this study shows us that there have been studies on Tessier craniofacial clefts numbers 3 and 4 as far back as 1980 [45]; however, evidence of the knowledge of anthropometry began in the year 2011. In addition, evidence from these studies indicate that the knowledge of anthropometry is key to a satisfactory outcome in the surgical management of these clefts; however, there is a scarcity of publication on this knowledge and this has made arriving at a consensus on standardized landmarks, reference measurements, and principles of repair difficult thus far. Also, evidence from these studies show that there is a generally acceptable knowledge of the morphology of a complete cleft of Tessier craniofacial clefts numbers 3 and 4; however, there is a sparsity of literature on the morphology of the incomplete numbers 3 and 4 Tessier craniofacial clefts. Although the problem of facial clefts is a global issue [4], this study further revealed that studies on the knowledge of anthropometry of Tessier craniofacial clefts numbers 3 and 4 were mostly done in middle-income countries (54.5%) while 45.5% was carried out in high-income countries, and none were done in low-income countries.

Cizmeci and Kuvat aimed at presenting a treatment option for these rare clefts and also reiterated that little is being published about the treatment and management of these clefts due to its rarity [20].

To the best of our knowledge, this is the first scoping review to map evidence on the knowledge of morphology and the knowledge of anthropometry in Tessier craniofacial clefts numbers 3 and 4. An extensive search strategy, which helped in the identification of a considerable number of studies, was conducted in this study. The study followed clear screening processes using keywords, which were guided by study PCC nomenclature. A thorough data search using Boolean terms was conducted during the literature search to increase the chances of finding eligible studies for inclusion in this review. The degree of agreement between the reviewers was significant (> 0.05) after the full-article screening. The review also included a transparent methodological quality assessment of the included primary studies using the recommended MMAT tool [12].

Despite the reported strength of our study, the limitation we encountered was primarily the inability to retrieve some articles which might have been of benefit to the study [13–17]. This was despite efforts including but not limited to personal letters to the authors. Also, Chen PK-T, et al. did not distinguish male from the female participants in their study [6].

Our review has shown that there is little in the form of research publications on the morphology (especially incomplete clefts) and more importantly on the anthropometry of Tessier craniofacial clefts numbers 3 and 4. We recommend to researchers that not only should more be done in documentation of incomplete clefts but more importantly research should be redirected mostly towards the emerging technique of anthropometry in the understanding and possibly finding a standardized way of managing the rare craniofacial clefts numbers 3 and 4. Also, we recommend that these studies require to be reported in low-resource countries as currently there is no evidence of such studies from these areas.

To surgeons in the management of these complex rare facial clefts, we recommend the use of anthropometric techniques in the way the repairs are carried out as this will prove to be a more reproducible method of repair and will further contribute to having a standardized way of carrying out these complex yet rewarding surgeries.

## Conclusion

Our findings suggest that the knowledge of morphology and the knowledge of anthropometry of Tessier craniofacial clefts numbers 3 and 4 exist albeit not fully harnessed. Furthermore, our review highlights the fact that no studies on these clefts are being done in low-income countries despite the global prevalence of this disease. This review also highlights the fact that knowledge of anthropometry is an emerging technique of solving the problem posed to surgeons of not having a standardized way of treating these defects. Further, studies should be encouraged in areas of anthropometry of Tessier craniofacial clefts numbers 3 and 4 as well as other aspects that affect the rare clefts Tessier numbers 3 and 4 such as their treatment, the outcome of this treatment, and possibly the clinical spectrum of their presentation.

## Additional files

Additional file 1: PRISMA checklist. (DOC 64 kb)

Additional file 2: Calculation of degree of agreement for full-article screening between the two reviewers. (PDF 86 kb)

## Abbreviations

MMAT: Mixed Method Appraisal Tool
PCC: Population, Concept, Context
PRISMA: Preferred Reporting Items for Systematic reviews and Meta-analyses
SDG: Sustainable Development Goals
USA: United States of America
WHO: World Health Organization
