# Supplementary material for: Scoping review of the morphology and anthropometry of Tessier craniofacial clefts numbers 3 and 4
Source: Syst Rev. 2019 Feb 4;8:42. doi: 10.1186/s13643-019-0951-6 (PMC6360760; doi:10.1186/s13643-019-0951-6)
Supplement: Supplementary file 2 — Calculation of degree of agreement for full-article screening between the two reviewers. (PDF 86 kb) [file 13643_2019_951_MOESM2_ESM.pdf]

Additional file 2:

List of studies included for full article screening and reviewers' responses

Author and Date	Response: Reviewer 1	Response: Reviewer 2
1. AbdollahiFakhim et al., 2013	Yes	Yes
2. Madaree et al., 1992	Yes	Yes
3. Horoz et al., 2016	Yes	Yes
4. Sigler et al., 2004	Yes	No
5. Morgan et al., 2016	Yes	Yes
6. Akoz et al., 1996	Yes	Yes
7. Maeda et al., 2014	Yes	Yes
8. Portier-Marret et al., 2008	Yes	Yes
9. Uemura et al., 2014	Yes	Yes
10. Bartlett et al., 2012	Yes	Yes
11. Sari et al., 2003	Yes	Yes
12. Valle et al., 2011	Yes	Yes
13. Rintala et al., 1980	Yes	Yes
14. Freitas et al., 2010	Yes	Yes
15. Resnick et al., 1990	Yes	No
16. Longaker et al., 1997	Yes	Yes
17. Chen et al., 2012	Yes	Yes
18. Giglio et al., 2008	Yes	Yes
19. Wu et al., 2013	Yes	Yes
20. Nor et al., 2016	Yes	Yes
21. Coruh et al., 2005	Yes	Yes
22. Sesenna et al., 2012	Yes	Yes
23. Spolyar et al., 2015	Yes	Yes

24. Laure et al., 2010	Yes	Yes
25. Alonso et al., 2008	Yes	Yes
26. Allam et al., 2014	Yes	Yes
27. Wenbim et al., 2007	Yes	Yes
28. Cizmeci et al., 2011	Yes	Yes
29. Alleman et al., 2016	Yes	No
30. Gawrych et al., 2010	Yes	Yes
31. Mishima et al., 1996	Yes	Yes
32. Tokioka et al., 2005	Yes	Yes
33. Balaji, S.M	Yes	Yes
34. Bodin et al., 2005	Yes	Yes
35. Darzi et al., 1993	Yes	Yes
36. Mishra et al., 2009	Yes	Yes
37. Rahpeyma et al., 2014	Yes	Yes
38. Xu et al., 2016	Yes	Yes
39. Reddy et al., 2014	Yes	Yes

```
kap  ResponseReviewer1 ResponseReviewer2

             Expected
Agreement   Agreement     Kappa   Std. Err.        Z      Prob>Z
-----------------------------------------------------------------
  92.31%      92.31%     0.0000     0.0000      0.00      0.5000

. mcc  ResponseReviewer1 ResponseReviewer2
```

```
                 | Controls               |
Cases            |   Exposed   Unexposed  |      Total
-----------------+------------------------+------------
         Exposed |       36          3    |         39
       Unexposed |        0          0    |          0
-----------------+------------------------+------------
           Total |       36          3    |         39

McNemar's chi2(1) =      3.00    Prob > chi2 = 0.0833
Exact McNemar significance probability       = 0.2500

Proportion with factor
        Cases             1
        Controls    .9230769     [95% Conf. Interval]
                   ---------     --------------------
        difference  .0769231     -.0323481   .1861943
        ratio       1.083333      .9894989   1.186066
        rel. diff.         1             1          1

        odds ratio         .      .4132314          .   (exact)
```
